# From By-Product to Bioactive Molecular Ingredient: The Impact of Cranberry Pomace on Antioxidant Properties and Enzyme Modulation in Functional Biscuits

**DOI:** 10.3390/ijms26189002

**Published:** 2025-09-16

**Authors:** Natalia Matłok, Tomasz Piechowiak, Ireneusz Kapusta, Maciej Balawejder

**Affiliations:** 1 Department of Food and Agriculture Production Engineering, University of Rzeszow, St. Zelwerowicza 4, 35-601 Rzeszow, Poland; 2 Department of Chemistry and Food Toxicology, University of Rzeszow, St. Ćwiklińskiej 1a, 35-601 Rzeszow, Poland; tpiechowiak@ur.edu.pl (T.P.); mbalawejder@ur.edu.pl (M.B.); 3 Department of Food Technology and Human Nutrition, College of Natural Sciences, University of Rzeszow, 4 Zelwerowicza St., 35-601 Rzeszow, Poland; ikapusta@ur.edu.pl

**Keywords:** cranberry pomace, polyphenols, non-extractable polyphenols (NEPP), antioxidant activity, functional foods, in vitro digestion, enzyme inhibition

## Abstract

Large-fruited cranberry (*Vaccinium macrocarpon* Ait.) is a rich source of polyphenolic compounds, including anthocyanins, flavonols, and unique A-type proanthocyanidins, which exhibit strong antioxidant and health-promoting properties. Cranberry pomace, a by-product generated during juice and concentrate production, remains underutilized despite being abundant in dietary fiber and non-extractable polyphenols (NEPPs). In this study, cranberry pomace was characterized phytochemically and applied as a functional ingredient in biscuits at levels of 5%, 10%, and 15% substitution of wheat flour. Total polyphenol content (TPC) and antioxidant activity (ABTS^•+^, DPPH^•^) were significantly higher in pomace compared to whole fruit values reported in the literature, which can be attributed to the concentration of polymeric proanthocyanidins and flavonols in skins and seeds. Biscuits enriched with pomace exhibited a dose-dependent increase in TPC and antioxidant capacity, with the 15% variant showing up to 6-fold higher polyphenol content and over 30-fold higher ABTS^•+^ activity after in vitro digestion compared to control. Digestion also released NEPP bound to the fiber matrix, improving bioaccessibility. Moreover, extracts from digested biscuits reduced oxidative stress in *Saccharomyces cerevisiae* and inhibited COX-1, COX-2, and AChE activities, suggesting potential anti-inflammatory and neuroprotective effects. These findings highlight cranberry pomace as a sustainable, high-value ingredient for functional foods, aligning with circular economy strategies.

## 1. Introduction

The American cranberry (*Vaccinium macrocarpon* Ait.) belongs to the *Ericaceae* family and is a species native to North America, now also cultivated in Europe, including Poland. This plant produces distinctive, spherical or slightly oval fruits of an intense red colour, typically ranging from 9 to 14 mm in diameter [1]. *V. macrocarpon* is a rich source of polyphenolic compounds, including anthocyanins, flavonols, and unique A-type proanthocyanidins (PAC-As), which exhibit a distinct structural profile compared to the more common B-type proanthocyanidins found in most fruits. In addition, the fruits contain organic acids (citric, malic, and benzoic acids), vitamin C (up to 15 mg/100 g of fresh weight), vitamin E, dietary fiber, and essential minerals such as manganese, copper, and potassium [2,3,4].

Due to their high acidity and polyphenol content, fresh American cranberries have limited shelf life in their unprocessed form. In the food industry, they are commonly processed into juices, concentrates, jams, dried fruits, and capsules containing cranberry extract [5].

Cranberry pomace, a by-product of *V. macrocarpon* processing, is generated primarily during juice and concentrate production. It results from mechanical pressing of the fruit, whereby the liquid phase (juice) is separated and the remaining solid residue constitutes the pomace. Although often regarded as post-production waste, cranberry pomace is a valuable source of dietary fiber and unique phenolic compounds. It has been demonstrated that, on a per-mass basis, berry pomace contains significantly higher concentrations of phytochemicals—particularly phenol compounds—than juice, and even the whole fruit. Effective utilisation of cranberry pomace may support the development of a circular economy and lead to the creation of high value-added products in the food, pharmaceutical, and cosmetic industries [6].

Recent studies have highlighted the growing interest in functional foods enriched with natural bioactive compounds due to their potential health benefits, including antioxidant, anti-inflammatory, and neuroprotective effects. Phenolic compounds, such as those abundant in cranberry pomace, have been linked to the prevention and management of chronic diseases, including neurodegenerative disorders like Alzheimer’s disease. Despite the well-documented health-promoting properties of cranberry polyphenols, there is limited research focusing on their stability, bioaccessibility, and biological activity when incorporated into processed foods, particularly bakery products subjected to heat treatment [7,8].

The present study aimed to evaluate the application potential of American cranberry pomace in the development of functional bakery products. Given that biscuits are a widely consumed sweet snack, shortcrust biscuits were formulated with the addition of freeze-dried *V. macrocarpon* pomace. These were then subjected to analyses of selected antioxidant and biological properties.

## 2. Results and Discussion

### 2.1. Phytochemical Characterisation of American Cranberry Pomace

The fruits of the American cranberry (*V. macrocarpon*) are a valuable raw material for the food industry, particularly in juice production, due to their high content of bioactive compounds such as phenol compounds, anthocyanins, and vitamin C. Juice pressing generates significant quantities of by-products, including pomace, which, despite the removal of the juice fraction, still retains substantial amounts of biologically active components and may be reused as a functional raw material [9].

In this study, pomace obtained from the pressing of V. macrocarpon fruits was subjected to phytochemical evaluation (Table 1). The total polyphenol content (TPC), determined using the Folin–Ciocalteu (FC) method, was 5.12 g GAE kg^−1^ dry matter. The total antioxidant activity was measured at 12.29 g TE kg^−1^ dry matter by the ABTS^•+^ method, and 6.38 g TE kg^−1^ dry matter by the DPPH^•^ assay. These elevated values confirm the strong antioxidant potential of cranberry pomace as a fraction rich in phenolic compounds. Notably, the TPC observed in the pomace was significantly higher than that reported in whole American cranberry fruits in the literature. Viskelis et al. [10], in their analysis of four cranberry cultivars, indicated that the maximum polyphenol content in the ‘Black Veil’ variety was approximately 5 g GAE kg^−1^. Similar findings were reported by Debnath and An [11], who showed that TPC in fresh cranberries generally ranges between 3–8 g GAE kg^−1^ fresh weight. Furthermore, Borowska et al. (2009) reported antioxidant activity values for large-fruited cranberry cultivars, with the ‘Pilgrim’ variety showing DPPH^•^ scavenging activity of 66 mmol TE per g of fresh mass and ABTS^•+^ activity of 9.3 mmol TE per g of fresh mass, which supports the high antioxidant potential of this species [3].

Due to its rich chemical composition, cranberry pomace can be utilised as a valuable raw material in further technological processes, such as a fortifying ingredient in food products (e.g., bakery goods), in the production of dietary supplements, functional extracts, or bioactive food additives. The observed higher polyphenol content in the pomace can be attributed to their concentration in the parts of the fruit richest in these compounds, namely the skins and seeds. Following juice extraction—primarily representing an aqueous extract of the pulp—the remaining solid matter consists predominantly of fruit components containing polymeric proanthocyanidins, flavonols, and anthocyanins bound to cell membranes, which contributes to the elevated TPC values.

This phenomenon was also evident during the qualitative and quantitative analysis of phenolic compounds in cranberry pomace (Table 1). The total phenolic content determined using the Folin–Ciocalteu spectrophotometric method was approximately twice as high as the sum of phenolic compounds identified using LC-MS. This discrepancy is likely due to the FC method’s analytical response to polymeric compounds with molecular weights that prevent chromatographic separation. These compounds most likely remain bound to the matrix and are not extracted into analytical samples for LC-MS analysis [12]. Importantly, these compounds may be released through depolymerisation processes during digestion, thereby increasing their bioavailability and enhancing the value of products containing them.

The predominant phenolic compound in cranberry pomace identified by LC-MS was quercetin 3-*O*-glucoside, which aligns with the phenolic profile previously established for cranberry fruit extracts [13].

Considering its favourable chemical profile, American cranberry pomace can be effectively utilised as a functional ingredient across various sectors of the food industry. Applications include use as an additive to enhance the nutritional value of baked goods, as a raw material for the production of functional extracts, and as a base for the development of dietary supplements or bioactive technological additives. Its revalorisation also supports strategies for sustainable development and a circular economy.

### 2.2. Phenolic Content and Antioxidant Activity of Enriched Biscuits

**Figure 1:**
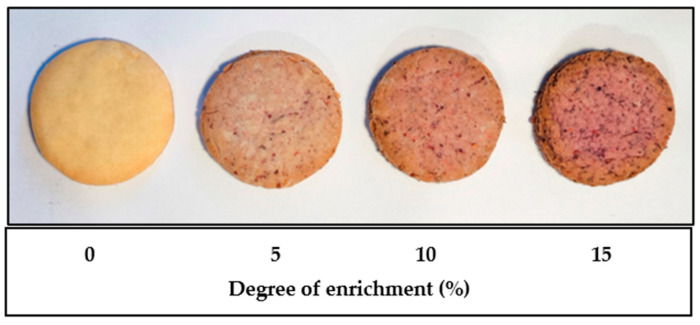
Functional biscuits supplemented with *V. macrocarpon* pomace.

As part of the study, biscuits were prepared with varying proportions of cranberry pomace, which was used to partially replace wheat flour. Four variants of baked products were developed, containing 0% (control), 5%, 10%, and 15% of pomace, calculated as a percentage of the flour weight (Figure 1).

**Figure 2:**
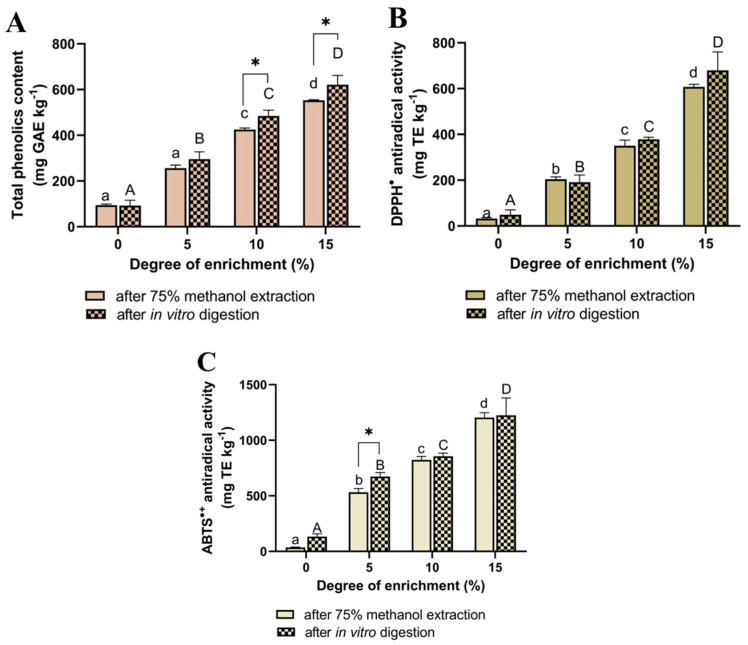
Total phenolic content (**A**) and antiradical activity against DPPH^•^ (**B**) and ABTS^•+^ (**C**) in biscuits enriched with cranberry pomace. For the samples extracted with 75% methanol, mean values marked with different lowercase letters are significantly different from each other, whereas for the in vitro digested samples, mean values marked with different uppercase letters differ significantly at α = 0.05. Mean values marked with * are significantly different from each other according to the results of Student’s test at α = 0.05.

The addition of cranberry pomace to bakery products significantly influenced their antioxidant potential. Changes in total polyphenol content (TPC) (Figure 2A) and antioxidant activity (Figure 2B,C) in the biscuits were correlated with the level of pomace enrichment. A clear trend was observed: increasing the proportion of pomace in the formulation led to a rise in both antioxidant activity and polyphenol content in samples measured before (extraction with 75% methanol) and after in vitro digestion.

The most pronounced effects were observed at the 15% enrichment level, where DPPH^•^ radical scavenging activity increased nearly 19-fold, and ABTS^•+^ activity increased 34-fold, in comparison to the control. This was accompanied by a 6-fold increase in total polyphenol content (Figure 2A–C).

Significantly higher TPC values were recorded for biscuits enriched with 10% and 15% pomace after in vitro digestion, compared to their respective TPC before digestion (extracted with 75% methanol). This increase is likely due to the presence of non-extractable polyphenols (NEPPs) in the cranberry pomace. These compounds are strongly bound to the dietary fiber matrix and are not detectable by standard analytical extraction methods [14,15]. However, during simulated in vitro enzymatic digestion, such compounds are released from the matrix, primarily through the hydrolysis of carbohydrates and proteins by digestive enzymes, thereby increasing the bioaccessible TPC [16].

A similar trend was observed in the antioxidant activity (DPPH^•^ and ABTS^•+^ assays) of bioaccessible compounds released from the biscuits during simulated intestinal digestion. This activity increased significantly with higher levels of pomace enrichment, which can be partly attributed to the enhanced release of specific polyphenolic compounds.

The effect of pomace addition on TPC and antioxidant activity after digestion varies depending on several factors, particularly the type of pomace (i.e., the fruit species from which it was obtained) and its physical structure—especially porosity, which facilitates polyphenol extraction. This was confirmed by Arcia et al. [17], who reported that TPC increased after digestion in apple and orange pomace, whereas a decrease was observed in grape pomace, most likely due to the destabilisation of anthocyanin.

During the digestion of food products, one of the main mechanisms involved is hydrolysis, which leads to the formation of low-molecular-weight compounds that are readily absorbed from the gastrointestinal tract [18]. Analysis of the phenolic profile in bakery products enriched with cranberry pomace revealed that the profile was analogous to that of the pomace itself, and the concentration of individual compounds was proportional to their content in the final product (Table 2).

However, the simulated in vitro digestion process significantly altered this profile, particularly through a marked reduction in phenolic glycosides. The concentration of the main compound, quercetin 3-*O*-glucoside, in the undigested product was almost 30 times higher than in the post-digestion fraction. It appears that during simulated in vitro digestion, aglycones also undergo degradation, as no corresponding increase in their levels was observed after digestion.

Nevertheless, it is important to note that the high content of glycosides and presumably polymeric phenolic fractions in the product contributes to a relatively high total phenolic content after digestion, which may enhance the value of such products as functional foods. This assumption is supported by the observed increase in TPC in the post-digestion fraction, as determined by the Folin–Ciocalteu method (Figure 2A).

It should be noted, however, that the Folin–Ciocalteu method is known to sometimes overestimate total phenolic content due to its nonspecific reaction with other reducing substances present in the sample, such as certain sugars, ascorbic acid, and aromatic amines. Moreover, some authors have used this method to assess antioxidant activity, which may contribute to variability in reported results. Addressing this limitation is important for the accurate interpretation of phenolic content and antioxidant capacity measurements in complex food matrices [18].

The antioxidant properties of the bioactive fraction from the biscuits were further confirmed through the assessment of oxidative stress levels in *Saccharomyces cerevisiae* cells exposed to hydrogen peroxide. This organism is widely used as a model in biological research due to the fact that certain redox homeostasis mechanisms are conserved and analogous to those found in higher organisms, including humans [13].

Due to the presence of hydrolysates of proteins, lipids, and polysaccharides in the post-digestion fraction, the extract was purified prior to application in the culture medium. This was achieved by removing ballast substances using a C18 column, thereby isolating the polyphenolic fraction.

**Figure 3:**
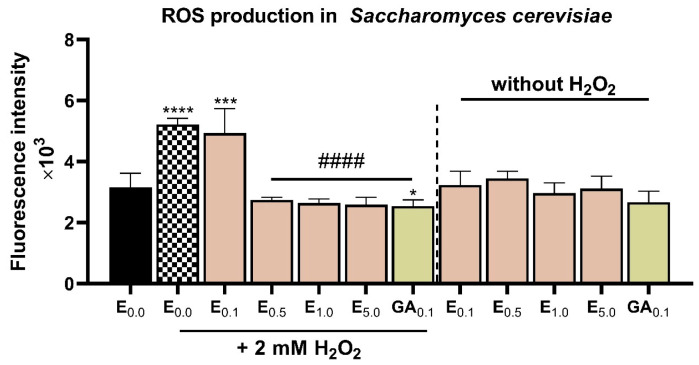
The effect of the bioactive (polyphenolic) fraction extracted from biscuits enriched with pomace (15%) previously subjected to in vitro digestion on the level of reactive oxygen species (ROS) in *S. cerevisiae* cells exposed to hydrogen peroxide. Mean values marked with *, *** or **** differ significantly from the control (untreated cells) at α = 0.05, 0.001, and 0.0001, respectively, according to the Tukey test. Mean values marked #### differ significantly from cells treated with H_2_O_2_ only, at α = 0.05, 0.01, 0.001 and 0.0001, respectively, according to the results of the Tukey test. In the figures: E—purified extract from biscuits at concentrations of 0 (control), 0.1, 1.0 and 5.0 mg mL^−1^. GA—gallic acid at a concentration of 0.1 mg mL^−1^.

As expected, the antioxidant fraction isolated from the 15% pomace-enriched biscuits (post-digestion) was capable of reducing oxidative stress in S. cerevisiae cells exposed to H_2_O_2_. As shown in Figure 3, incubation of yeast cells with 2 mM H_2_O_2_ led to a 66% increase in the production of reactive oxygen species (ROS) compared to the control. Application of the extract at concentrations ranging from 0.5 to 5 mg mL^−1^ prior to H_2_O_2_ exposure mitigated the pro-oxidative effect. At the highest concentration, the extract reduced ROS generation by 50% relative to the positive control (cells treated with H_2_O_2_ but without the extract). This effect was comparable to the antioxidant activity of gallic acid at a concentration of 0.1 mg mL^−1^.

Importantly, when administered alone, without H_2_O_2_, the extract did not induce ROS production in the cells, similar to gallic acid. These findings are consistent with the results reported by Balawejder et al., who demonstrated the antioxidant activity of an ethanolic extract from whole American cranberries in yeast cells also subjected to hydrogen peroxide-induced oxidative stress. In their study, the presence of an extract—rich in quercetin 3-*O*-glucoside, peonidin 3-*O*-glucoside, and quercetin 3-*O*-pentoside—at a concentration of 50 µg mL^−1^ in the culture reduced ROS formation by 30% compared to H_2_O_2_-treated cells [13].

These observations suggest that the consumption of products enriched with American cranberry pomace, such as the formulated biscuits, may help reduce cellular stress in organisms beyond the *S. cerevisiae* model.

The polyphenol fraction obtained from the biscuits after in vitro digestion also exhibited the ability to inhibit the activity of enzymes associated with neurodegenerative processes, namely acetylcholinesterase (AChE), cyclooxygenase-1 (COX-1), and cyclooxygenase-2 (COX-2). AChE inhibitors are used to enhance neurotransmission and improve cognitive function in disorders such as Alzheimer’s disease and dementia [19,20,21]. On the other hand, COX inhibitors reduce inflammation in cells, which is one of the contributing factors to neuronal damage.

**Figure 4:**
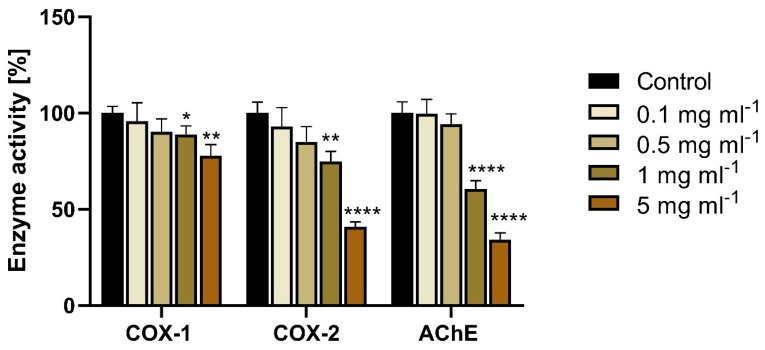
The effect of the bioactive (polyphenolic) fraction extracted from cookies previously digested in vitro on the activity of cyclooxygenase 1 (COX-1), cyclooxygenase 2 (COX-2) and acetylcholinesterase (AChE). Mean values marked with *, **, and **** differ significantly from the control at α = 0.05, 0.01, and 0.0001, respectively, according to the results of the Tukey test.

The polyphenol fraction derived from the pomace-enriched biscuits showed a clear inhibitory effect on the tested enzymes. A 50% inhibition of enzymatic activity was observed for both AChE and COX-2 at a concentration of 5 mg mL^−1^. At the highest tested concentration, AChE activity was reduced by 66%, and COX-2 activity by 59% (Figure 4).

Similar to the findings presented in this study, Balawejder et al. [13] previously reported significant AChE and COX-2 inhibitory activity of polyphenols from American cranberry in the same enzymatic systems. The ability of polyphenols to inhibit AChE activity has also been demonstrated for extracts from açaí berries, bilberries, blueberries, grape skins, and paper mulberry. In turn, anthocyanins derived from cranberry, blueberry, bilberry, elderberry, strawberry, raspberry, and blackberry have been shown to inhibit COX-1 and COX-2.

## 3. Materials and Methods

### 3.1. Preparation of Fruit Pomace

Fruit pomace was obtained from cranberry fruit using a slow-speed juicer. The pomace was immediately frozen at −67 °C and then freeze-dried using a Harvest Right freeze dryer (Salt Lake City, UT, USA) at a pressure of 20 Pa and a shelf temperature of 35 °C. After drying, the pomace was ground using a laboratory powder mill and used for further processing.

### 3.2. Biscuits Preparation

Biscuits were prepared by first mixing 50 g of butter (82% fat) with 75 g of chicken eggs and 75 g of beet sugar using a planetary mixer (Kenwood, Hertfordshire, United Kingdom). Subsequently, 4 g of baking powder and 300 g of wheat flour (type 450) were added to the mixture. The dough was thoroughly mixed, rolled out to a thickness of 0.5 cm, and cut into 4 cm diameter biscuits. The biscuits were baked at 190 °C for 12 min and then cooled to room temperature. Biscuits with pomace were prepared in the same manner, with the exception that 5%, 10%, or 15% of the wheat flour was replaced with ground cranberry pomace. Prior to analysis, all biscuits were ground into a fine powder.

### 3.3. Analysis of Total Antioxidants Activity

Antioxidants were extracted from 5 g of biscuit powder and 0.5 g of pomace using 20 mL of 75% methanol. The samples were homogenized, shaken on an orbital shaker for 1 h and 30 min, and centrifuged at 15,000× *g* for 30 min. The resulting supernatant was collected for analysis. The antioxidant activity was assessed using ABTS^•+^ and DPPH^•^ radical scavenging assays. Total polyphenol content was determined using the Folin–Ciocalteu method (FC), following the protocols described by Piechowiak and Sowa-Borowiec [22]. Antioxidant activity was expressed as Trolox equivalents, and total polyphenol content as gallic acid equivalents per kilogram of material.

### 3.4. Extraction Procedure and Determination of Individual Polyphenols

Extracts were prepared from dried, ground material. Briefly, 1 g of ground material was combined with 10 mL of 50% methanol in water (*v*/*v*) and sonicated (ultrasonic bath, Sonic 10, Polsonic, Warsaw, Poland) for 20 min at 30 °C. The suspension was centrifuged (Centrifuge 5430, Eppendorf, Hamburg, Germany), the supernatant was collected, and the residue was resubjected to the above extraction. After combining the centrifuged extracts, the methanol was evaporated in a rotary evaporator (R-215 Rotavapor System, Buchi, Flawil, Switzerland) at 40 °C. Concentrated samples were applied to a solid-phase extraction (SPE) C18 Sep-Pak cartridge (Waters Associates, Milford, MA, USA) preconditioned with water. The cartridge was washed first with water to remove sugars. Phenolics were eluted with 40% MeOH, evaporated, and redissolved in MeOH (1 mL) For UPLC, 150 µL was transferred to another vial, and the solution was made up to a final volume of 1.5 mL with MeOH.

The identification of individual polyphenolic compounds was carried out using the UPLC-PDA-MS method according to the procedure of Balawejder et al. [23]. In detail: for UPLC-PDA-MS/MS analysis, anthocyanins were analyzed using a Waters ACQUITY system (Waters, Milford, MA, USA), consisting of a binary pump manager, sample manager, column manager, PDA detector and tandem quadrupole mass spectrometer (TQD) with electrospray ionization (ESI). The separation was carried out using BEH C18 column (100 mm × 2.1 mm i.d., 1.7 µm, Waters) kept at 50 °C. The mobile phase A (2% formic acid in water *v*/*v*) and B (2% formic acid in 40% ACN in water *v*/*v*) were prepared. The gradient program was set as follows: 0 min 5% B, from 0 to 8 min linear to 100% B, and from 8 to 9.5 min for washing and back to initial conditions. The injection volume of the samples was 5 µL (partial loop with needle overfill) and the flow rate was 0.35 mL/ min. The following parameters were used for TQD: capillary voltage 3.5 kV; con voltage 30 V in positive mode, the source was kept at 250 °C and desolvation temperature was 350 °C; con gas flow of 100 L/h; and desolvation gas flow of 800 L/h. Argon was used as the collision gas at a flow rate of 0.3 mL/min. The anthocyanin detection and identification were based on specific PDA spectra, mass to charge ratio and fragment ions obtained after collision-induced dissociation (CID). The quantitative analysis was based on specific MS transitions in multiple reaction monitoring (MRM) mode. The MRM transitions, cone voltage and collision energy of each individual anthocyanins were set manually with a dwell time of at least 25 ms. Further settings are listed in Table 2. All determinations were performed in duplicate. Waters MassLynx software v.4.1 was used for data acquisition and processing.

### 3.5. In Vitro Digestion of Biscuits

In vitro digestion was performed on both control biscuits and biscuits containing 15% pomace, according to the procedure by Złotek et al. (2017) [24] with slight modifications. A 7.0 g sample of ground biscuits was weighed into conical flasks and mixed with 15 mL of simulated saliva solution containing 2.38 g L^−1^ Na_2_HPO_4_, 0.19 g L^−1^ KH_2_PO_4_, 8 g L^−1^ NaCl, 100 mg L^−1^ mucin, and 200 U mL^−1^ α-amylase. The mixture was shaken at 37 °C for 10 min. Subsequently, 15 mL of simulated gastric juice (pH 1.2) containing 300 U mL^−1^ pepsin and 0.03 M NaCl was added. The mixture was incubated for 60 min at 37 °C, then neutralized to pH 7.0 using 1 M NaOH. Following this, 5 mL of 120 mmol L^−1^ NaCl and 120 mmol L^−1^ KCl, along with 5 mL of simulated intestinal fluid (containing 0.05 g pancreatin and 0.3 g bile dissolved in 35 mL of 0.1 M NaHCO_3_), were sequentially added. The samples were incubated for 2 h at 37 °C. The digested fraction was centrifuged at 10,000× *g* for 30 min at 4 °C. Before analysis, the supernatant was diluted 1:1 (*v*/*v*) with methanol and centrifuged again under the same conditions. Antioxidant activity (ABTS^•+^, DPPH^•^) and total polyphenol content were determined in the final supernatant.

### 3.6. Assessment of Antioxidant Properties Using Saccharomyces Cerevisiae

To evaluate the antioxidant activity of the bioactive fraction from cranberry pomace-enriched biscuits, ballast components (sugars, fats, protein hydrolysates and enzymes) were first removed from the post-digestion supernatant. This was achieved using Sep-Pak C18 silica-filled solid-phase extraction columns (360 mg, 55–105 μm; Waters, Milford, MA, USA). Columns were pre-conditioned by washing three times with distilled water, followed by three washes with methanol. Then, 5 mL of the digested supernatant was applied to the column. The column was washed three times with distilled water, and antioxidants were eluted twice with methanol. The methanol eluate was evaporated using a rotary evaporator, and the dry residue was reconstituted in phosphate-buffered saline (PBS, pH 7.4) to a final concentration of 5 mg mL^−1^. The extract was then diluted with PBS to concentrations of 1.0, 0.5, and 0.1 mg mL^−1^ for further analysis.

*Saccharomyces cerevisiae* yeast (NCPF 3178) was cultured at 28 °C with shaking (150 rpm) in a medium containing 1% yeast extract, 1% peptone, and 2% glucose, starting at an initial optical density of A_600_ = 0.01. The culture was harvested during the logarithmic growth phase (after 18 h), diluted to A_600_ = 0.5, and transferred into 12-well plates (1.8 mL per well). Then, 100 µL of each extract or PBS (control) was added. The yeast was incubated for 2 h at 28 °C with shaking (180 rpm), centrifuged (4000× *g*, 5 min), washed with fresh medium, and resuspended in 1.8 mL of fresh medium. Galic acid (0.1 mg mL^−1^) was used as a positive control with a protective effect.

For the oxidative stress induction, 100 µL of H_2_O_2_ (in PBS) was added to achieve a final concentration of 2 mM (PBS was added to the control). After 1 h of incubation, the yeast was centrifuged again, washed twice with PBS (containing 1% glucose), and resuspended in 1 mL of PBS with 1% glucose and 10 µM H_2_DCF-DA. Following 1 h of incubation in the dark with shaking, fluorescence was measured (excitation: 495 nm; emission: 525 nm) using a Hitachi F7000 spectrofluorometer (Tokyo, Japan).

### 3.7. Inhibition of COX-1, COX-2, and AChE

The inhibitory activity of the digested fractions was evaluated against cyclooxygenase-1 (COX-1), cyclooxygenase-2 (COX-2), and acetylcholinesterase (AChE). COX-1 and COX-2 activity was determined using commercial assay kits from Cayman Chemical (cat. no. 701070 and 701080). AChE inhibition was measured using a Sigma-Aldrich (Saint Louis, MO, USA) assay (cat. no. MAK324). Inhibition of COX-1, COX-2 and ACHE was determined following the methodology described in the study by Balawejder et al. [13].

### 3.8. Statistical Analysis

The significance of differences between three or more means was tested using ANOVA analysis and Tukey’s test, while differences between two means were tested using Student’s *t*-test. Significance levels of α = 0.05, 0.01, 0.001, and 0.0001 were used. Statistical analysis was performed in GraphPad 8.0.

## 4. Conclusions

The results of this study clearly indicate that cranberry pomace is a rich source of polyphenols and can be successfully valorized as a functional food ingredient. The total polyphenol content (TPC) in cranberry pomace reached values nearly twice as high as the sum of individual compounds identified by LC–MS, which can be attributed to the presence of non-extractable polyphenols (NEPPs) bound to the fiber matrix. Incorporation of pomace into biscuits significantly increased their biological value: the variant with 15% pomace addition exhibited a 6-fold higher TPC, a 19-fold increase in DPPH^•^ scavenging activity, and a 34-fold increase in ABTS^•+^ radical scavenging activity after in vitro digestion compared to the control. Moreover, digestion enhanced the release of bound phenolics, confirming the improved bioaccessibility of these compounds. Bioactive fractions from digested biscuits reduced ROS generation in *Saccharomyces cerevisiae* exposed to H_2_O_2_ by nearly 50% at the highest tested concentration (5 mg·mL^−1^) and showed enzyme inhibitory effects, lowering COX-1 activity by 22%, COX-2 by 59%, and AChE by 66%. These quantitative findings support the conclusion that cranberry pomace can substantially enrich bakery products with bioactive compounds, providing both antioxidant and enzyme-modulatory benefits. The incorporation of pomace into food matrices thus represents not only a sustainable approach to agro-industrial waste management but also a strategy for developing next-generation functional foods with proven health-promoting potential. In the future, a product with enhanced health benefits could be considered by eliminating sugar from the matrix or replacing it with a substitute, e.g., stevia.

## Figures and Tables

**Table 1:** Basic phytochemical characteristics of cranberry pomace.

Compound		Rt (min)	λ_max_	MS	MS/MS	Content (mg kg^−1^)
**Anthocyanins**						
**1.**	Cyanidin 3-*O*-glucoside	2.54	279, 512	449^+^	287	598.08 ± 12.68
**2.**	Cyanidin 3-*O*-galactoside	2.68	279, 517	449^+^	287	10.75 ± 0.13
**3.**	Cyanidin 3-*O*-arabinoside	2.86	279, 512	419^+^	287	375.27 ± 3.65
**4.**	Peonidin 3-*O*-glucoside	3.11	279, 512	463^+^	301	795.91 ± 72.01
**5.**	Peonidin 3-*O*-galactoside	3.27	278, 519	463^+^	301	44.61 ± 0.10
**6.**	Peonidin 3-*O*-arabinoside	3.45	279, 517	433^+^	301	266.68 ± 9.84
**Other phenolics**						
**7.**	Caffeic acid *O*-glucoside	2.40	329	341^−^	179	5.17 ± 0.01
**8.**	3-*O*-Caffeoylquinic acid	2.79	324	353^−^	191	20.03 ± 2.07
**9.**	Coumaric acid *O*-glucoside	2.96	310	325^−^	163	33.79 ± 0.98
**10.**	3-*O*-Feruloylquinic acid	3.32	329	355^−^	193	19.36 ± 0.41
**11.**	Sinapic acid 3-*O*-glucoside	3.39	329	385^−^	223	33.30 ± 5.61
**12.**	Myricetin 3-*O*-glucoside	3.78	257, 355	479^−^	317	120.90 ± 12.51
**13.**	Myricetin 3-*O*-arabinofuranoside	4.23	257, 352	449^−^	317	26.96 ± 0.26
**14.**	Quercetin 3-*O*-glucoside	4.40	255, 355	463^−^	301	186.79 ± 16.90
**15.**	Laricitrin 3-*O*-glucoside	4.51	271, 354	493^−^	331	19.23 ± 0.04
**16.**	Quercetin 3-*O*-xylopyranoside	4.72	255, 355	433^−^	301	18.03 ± 0.67
**17.**	Coumaroyl-dihydromonotropein	4.91	310	537^−^	163, 119	17.53 ± 0.02
**18.**	Quercetin 3-*O*-arabinopyranoside	4.96	255, 355	433^−^	301	65.31 ± 6.73
**19.**	Quercetin 3-*O*-rhamnoside	5.11	255, 352	447^−^	301	31.83 ± 0.93
**20.**	Syringetin 3-*O*-glucoside	5.17	271, 352	507^−^	345	16.53 ± 0.35
**21.**	Kaempferol 3-*O*-glucoside	5.64	267, 352	447^−^	285	6.44 ± 1.08
**22.**	Kaempferol 3-*O*-galactoside	5.76	265, 350	447^−^	285	4.84 ± 0.50
**23.**	Syringetin 3-*O*-pentoside	5.89	271, 350	477^−^	345	4.54 ± 0.11
**Sum of polyphenols identified by LC-MS (mg kg^−1^)**						2721.89 ± 147.62
**Total phenolics content (g GAE kg^−1^)**						5.12 ± 0.51
**Antiradical activity against DPPH^•^ (g TE kg^−1)^**						6.38 ± 1.96
**Antiradical activity against ABTS^•+^ (g TE kg^−1^)**						12.29 ± 1.88

**Table 2:** Polyphenol profile in biscuits enriched with cranberry pomace (15%) before and after in vitro digestion.

Compound		Content (mg kg^−1^)	
		Biscuits	Biscuits Subjected to In Vitro Digestion
**Anthocyanins**			
**1.**	Cyanidin 3-*O*-glucoside	48.75 ± 1.03 *	15.65 ± 1.55 *
**2.**	Cyanidin 3-*O*-galactoside	2.02 ± 0.03 *	1.39 ± 0.03 *
**3.**	Cyanidin 3-*O*-arabinoside	25.20 ± 0.25 *	6.51 ± 0.28 *
**4.**	Peonidin 3-*O*-glucoside	52.54 ± 4.75 *	21.39 ± 0.41 *
**5.**	Peonidin 3-*O*-galactoside	3.57 ± 0.01 *	1.28 ± 0.04 *
**6.**	Peonidin 3-*O*-arabinoside	16.59 ± 0.61 *	5.32 ± 0.33 *
**Other phenolics**			
**7.**	Caffeic acid *O*-glucoside	1.95 ± 0.00	1.05 ± 0.11
**8.**	3-*O*-Caffeoylquinic acid	7.48 ± 0.77	7.17 ± 0.35
**9.**	Coumaric acid *O*-glucoside	8.46 ± 0.25 *	5.12 ± 0.15 *
**10.**	3-*O*-Feruloylquinic acid	5.91 ± 0.13	6.79 ± 0.12
**11.**	Sinapic acid 3-*O*-glucoside	6.03 ± 1.02 *	4.10 ± 0.01 *
**12.**	Myricetin 3-*O*-glucoside	36.94 ± 3.82 *	4.22 ± 0.10 *
**13.**	Myricetin 3-*O*-arabinofuranoside	10.25 ± 1.02 *	6.12 ± 0.13 *
**14.**	Quercetin 3-*O*-glucoside	64.71 ± 1.42 *	2.20 ± 0.03 *
**15.**	Laricitrin 3-*O*-glucoside	11.31 ± 0.48	13.61 ± 0.13
**16.**	Quercetin 3-*O*-xylopyranoside	6.82 ± 0.13 *	4.46 ± 0.40 *
**17.**	Coumaroyl-dihydromonotropein	7.49 ± 0.26 *	1.94 ± 0.00 *
**18.**	Quercetin 3-*O*-arabinopyranoside	20.30 ± 1.25 *	0.45 ± 0.02 *
**19.**	Quercetin 3-*O*-rhamnoside	11.70 ± 1.24 *	1.76 ± 0.00 *
**20.**	Syringetin 3-*O*-glucoside	6.47 ± 0.32 *	1.91 ± 0.20 *
**21.**	Kaempferol 3-*O*-glucoside	1.88 ± 0.06	<LOQ
**22.**	Kaempferol 3-*O*-galactoside	1.76 ± 0.03	<LOQ
**23.**	Syringetin 3-*O*-pentoside	1.68 ± 0.01	<LOQ
**Sum of polyphenols identified by LC-MS (mg kg^−1^)**		359.81 ± 18.88	112.45 ± 4.41

Note: Statistically significant differences between the control and cranberry pomace-enriched variants are indicated by *.

## Data Availability

The original contributions presented in this study are included in the article. Further inquiries can be directed to the corresponding author.
